# Successful retrieval of deep intracardiac migrated broken umbilical venous catheter in a preterm infant: Case report

**DOI:** 10.1016/j.radcr.2023.12.022

**Published:** 2024-01-13

**Authors:** Abubakr Bajaber, Magda Hag Ali, Adeeb Omar Bazuhair, Omar Bajaber, Moath Alsaiady, Samy Rabie, Latifa BinMahmoud, Doaa Alfaki

**Affiliations:** a College of Medicine, Alfaisal University, Riyadh 11533, Saudi Arabia; b Department of Pediatric Cardiology, King Saud Medical City, Riyadh 12746, Saudi Arabia; c Medical Imaging Department, Interventional Radiology Section, King Saud Medical City, Riyadh 12746, Saudi Arabia; d Medical Imaging Department, Pediatric Radiology Section, King Saud Medical City, Riyadh 12746, Saudi Arabia; e Neonatal Critical Care Department, King Saud Medical City, Riyadh 12746, Saudi Arabia

**Keywords:** Umbilical catheter, Broken, Foreign body retrieval, Interventional radiology, Preterm infant, Heart

## Abstract

Umbilical catheters serve as indispensable tools in the realm of neonatal intensive care, contributing significantly to the well-being of premature infants. While rare, it is essential to approach their handling with utmost caution, as it can lead to fatal complications. We report a case of a preterm 9-day-old male infant, who was referred to our center for specialized treatment following an unsuccessful surgical attempt to address a fractured umbilical venous catheter (UVC). This case underscores the value of employing imaging techniques for prompt identification of such complications. Furthermore, the utilization of endovascular therapy emerges as a promising intervention in managing such complexities, thereby expanding the horizons of interventional radiology in elevating the standard of patient care.

## Introduction

Umbilical catheters, whether arterial or venous, are a vital measure implemented for infants admitted to the neonatal intensive care unit (NICU), providing crucial monitoring, and facilitating the administration of fluids, nutrition, and medications. However, if not handled with utmost care, they can lead to rare but potentially critical complications in this fragile population. The available literature on this subject remains limited, highlighting the need for further research to comprehensively grasp this issue, aiming at its prevention and the development of optimal management strategies. Herein, we present a case involving a preterm 9-day-old male infant whose umbilical venous catheter (UVC) broke and dislodged deeply in the left atrium.

## Case presentation

A male infant, born prematurely at 35 weeks of gestation, who is 9 days old and weighs 2400 grams, was transferred to our hospital for care. He was delivered via emergency lower segment cesarean section and initially admitted to the NICU at the referring hospital due to respiratory distress and acidosis. The infant required mechanical ventilation and a UVC was inserted.

On the eighth day of life, the infant's condition stabilized, and he was successfully weaned off the ventilator. However, during an attempt to remove the UVC, it was discovered that the catheter had broken and become dislodged into a deep position, making it impossible to visualize. Efforts by a pediatric surgeon to remove it through small surgical incision were unsuccessful. Due to limited resources for further catheter removal at the referring hospital, the decision was made to transfer the patient to our hospital for specialized care.

**Fig. 1:**
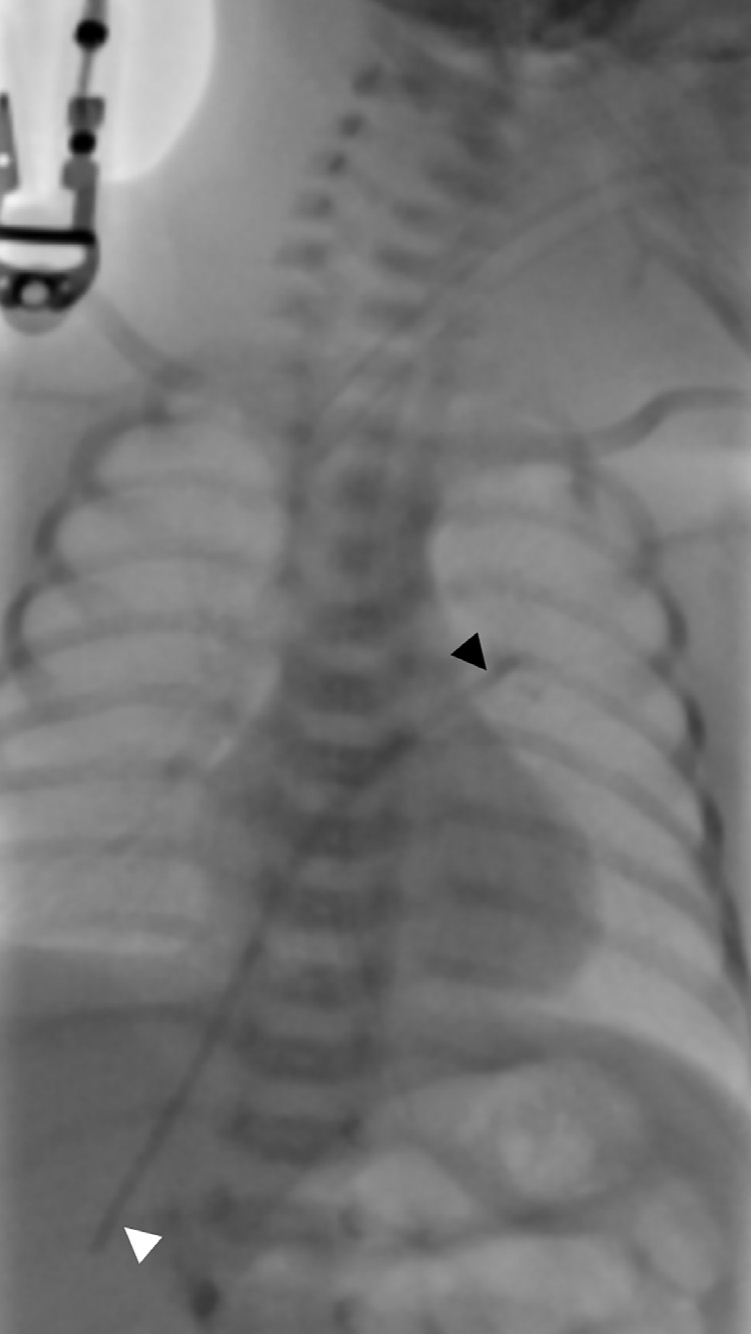
Posteroanterior view of the chest shows a retained catheter fragment extending from the hepatic vein (white arrowhead) to the left upper pulmonary vein (black arrowhead).

**Fig. 2:**
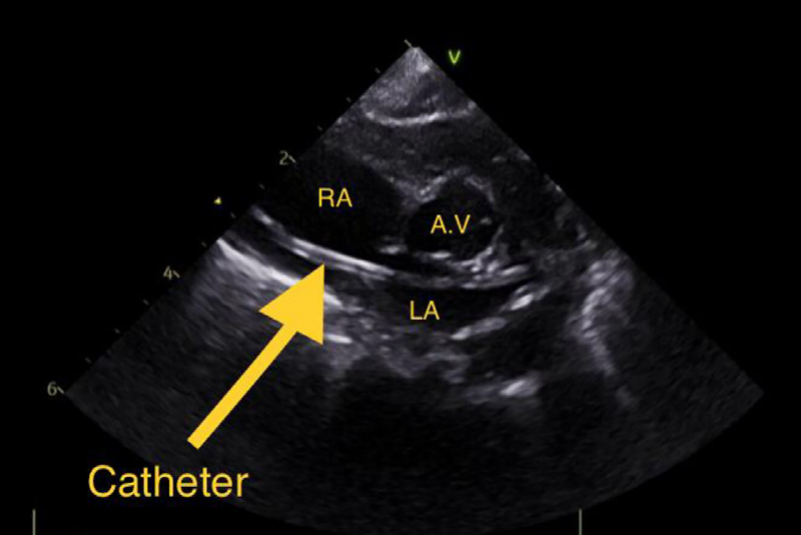
Parasternal short axis view of the transthoracic echocardiography shows the catheter (arrow) in the right atrium passing through patent foramen ovale into the left atrium. RA: Right atrium; LA: Left atrium; A. V: Aortic valve.

**Fig. 3:**
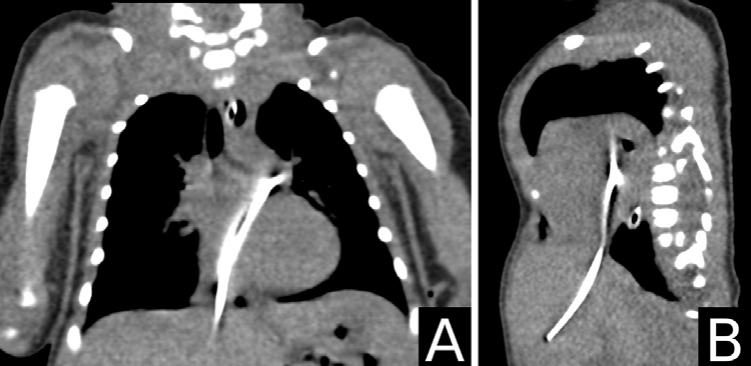
Coronal (A) and sagittal (B) reconstructed computed tomography (CT) images show a clear view of an opaque cylindrical foreign body representing the retained catheter fragment.

Upon admission to our NICU, the infant received care in an incubator and was provided with 2 liters of oxygen via nasal cannula. His vital signs were stable, and all laboratory tests were within normal limits, except for a mild elevation in serum bilirubin (Total: 218.1 µmol/L and direct: 24.8 µmol/L). The dislodgment and migration of the UVC into an intracardiac position were initially detected through a chest X-ray (Fig. 1). This was subsequently confirmed by transthoracic echocardiography (Fig. 2) and computed tomography (Fig. 3) which showed the catheter fragment traversing from the right atrium to the left atrium through a foramen ovale, ultimately reaching the left upper pulmonary vein.

**Fig. 4:**
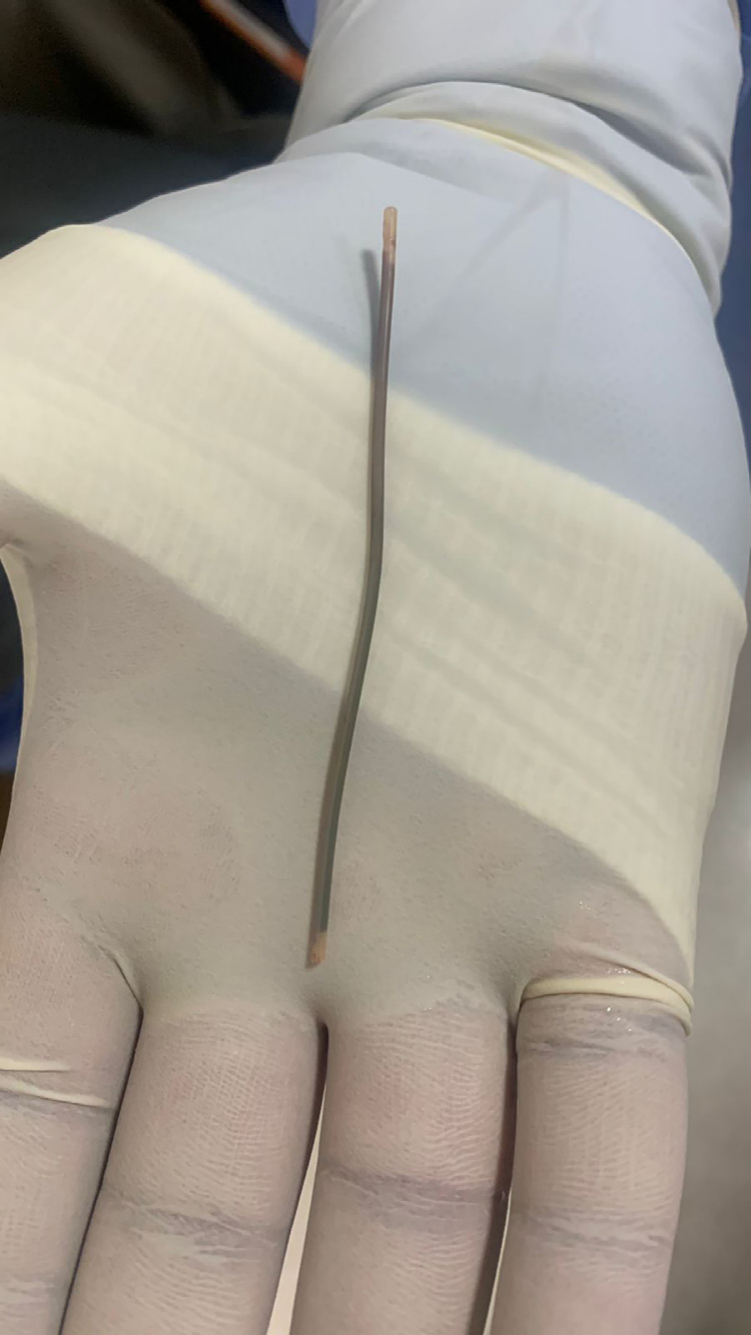
Gross view of the retrieved catheter fragment.

Our interventional radiology service was consulted, and the decision was made to schedule an emergency procedure for the removal of the foreign body on the infant's tenth day of life. The patient was prepared and draped in the standard sterile fashion; however, heparinization was omitted due to the hematologist's assessment that the potential risk of bleeding outweighed the benefits. Under general anesthesia, attempts were made to snare the catheter percutaneously through abdominal wall over the liver using micro alligator forceps to access the hepatic vein, but these efforts were unsuccessful. Subsequently, the right femoral vein was punctured under ultrasound guidance, and a 6 Fr sheath (Cordis Corporation; Florida, USA) was inserted. Using a single-loop 6 Fr snare (Merit Medical; Utah, USA), the dislodged catheter fragment (Fig. 4) was successfully retrieved from the left pulmonary vein through the patent foramen ovale (Video 1). Postremoval venacavography showed complete retrieval of the foreign body with no abnormal findings, and no immediate complications were observed.

## Discussion

Umbilical catheters are a standard element of protocols in NICU, frequently used to establish temporary central line access until a more permanent alternative can be arranged, if necessary. Although rare, complications associated with these catheters can have life-threatening consequences if not promptly addressed. The cumulative complication rate is notably higher in the UVC population, at 13.3%, compared to the Umbilical Arterial Catheter (UAC) population, which stands at 2.5%. However, it is important to note that complications tend to manifest earlier in patients with UAC [1].

These complications encompass catheter breakage, malpositioning of the catheter tip, bloodstream infections, effusions, and thrombosis. Notably, catheter breakage is a rare occurrence, observed in only 1% of patients with UAC-dwelled catheters and even less frequently in UVC-dwelled patients, occurring in only 0.4% of cases [1].

The duration of catheter placement is the most significant factor associated with a higher cumulative incidence of complications [1]. Hence, early removal of the catheter is recommended. In our case, the catheter was inserted and secured without any notable incidents, and its duration was not extended. However, complications arose during its removal. This underscores the human factor in these complications and highlights the necessity for the expertise of highly specialized practitioners to perform such instrumental procedures in a sterile setting, reducing the odds of complications.

Based on Habib et al. literature review, only a limited number of cases have been documented regarding this particular complication [2]. Different methods have been employed to address the issue of broken umbilical catheters. While an open surgical approach has been attempted in some instances, it has proven ineffective in certain reported cases as in our case [3,4]. Nevertheless, this approach becomes more feasible when dealing with a retained catheter fragment situated closer to the skin, nearer to the umbilicus [5].

Dhua et al. noted that the unsuccessful outcome of this approach might have been caused by the absence of vascular clamping before surgical intervention [6]. The purpose of clamping was to prevent further emigration of the catheter and enhance the likelihood of successful retrieval. However, in a separate instance, other team did employ clamping, but their retrieval efforts were still unsuccessful [3].

On the contrary, the fluoroscopy-guided endovascular approach is gaining popularity and has demonstrated higher success rates, even in those failing surgical management as in our case [2]. Nonetheless, in extremely low birth weight (ELBW) neonates (<1000 gm), the nonsurgical approach may present challenges. This is because they often require smaller diameter interventional tools (≤ 5 Fr), making the transcatheter foreign body removal a more intricate and dexterous procedure [3]. Despite these challenges, the nonsurgical approach holds significant promise, particularly in this vulnerable patient population.

Various endovascular techniques have been documented, with snaring emerging as the most reported method [2]. The choice of the route (eg, umbilical or femoral) employed depends on the specific location of the embolized catheter [7]. Similar to our case, some patients have necessitated transfer to a specialized center for optimal care in managing this complication [2,7]. This requirement for access to a specialized center can present a significant challenge, particularly in regions with limited resources and challenging logistics for patient transfers. This underscores the imperative for more robust preventive measures to mitigate the occurrence of such complications.

The implementation of guidelines governing the use of umbilical catheters in the care of NICU patients holds the potential to standardize the application of this intervention, thus reducing the risk of unfavorable outcomes [8]. Additionally, it is crucial to adopt appropriate precautions, as inadvertent catheter damage can occur due to sharp tools (such as needles or blades) or tightened suturing during catheter insertion, fixation, or removal, particularly given the challenge of restraining neonates [9]. A best practice entails a meticulous assessment of catheters’ length for potential transections after their removal, employing imaging to eliminate the possibility of retained fragments as clinical presentation can be asymptomatic or misleading in certain cases [2,7,10,11]. In case of suspicion, the use of imaging becomes imperative for a thorough investigation of foreign bodies, ensuring timely diagnosis, as delayed identification could result in a more complex clinical course [10].

## Conclusion

In conclusion, umbilical catheters are crucial life-saving interventions for critically ill neonates, albeit posing rare life-threatening risks. Consequently, their management by highly skilled specialists adhering to established guidelines is imperative to mitigate complications. Given that retained fragments can initially be asymptomatic or present with misleading clinical features, imaging plays a pivotal role in investigating these cases to avert more complex outcomes. While limited literature examines UVC breakage and its remedies, including our own case report, an endovascular approach has emerged as a promising option, boasting a higher success rate and lower complication rate, particularly among this vulnerable population. Further research is warranted to comprehensively explore the potential of interventional radiology in addressing such cases, thereby enhancing our understanding and refining clinical practices in neonatal care.

## Patient consent

An informed consent was taken from the patient's parent for the publication of this case report.

## Authors’ contributions

A.B. drafted the manuscript; M.H.A., A.O.B., and O.B. revised and edited the manuscript; S.R., L.B., and D.A. provided the clinical data; M.A. provided the imaging findings. All authors reviewed the final version of the manuscript and approved its submission.

## Appendix Supplementary materials

**Video (1):** Fluoroscopy clip shows a single-looped snare advanced through the inferior vena cava into the right atrium, and then, utilizing the patent foramen ovale, the snare was further guided into the left atrium. The distal tip of the catheter fragment was captured within the snare, and the entire fragment was safely retrieved. Image, video 1
